# Supplementary material for: WordCommentsAnalyzer: A windows software tool for qualitative research
Source: F1000Res. 2018 Sep 4;7:536. Originally published 2018 May 3. [Version 2] doi: 10.12688/f1000research.14819.2 (PMC6137411; doi:10.12688/f1000research.14819.2)
Supplement: Supplementary file 3 [file f1000research-7-17614-s0002.tgz › b2a79496-4ada-47c4-9682-a99ce867836d.zip!/Eight Iranian Health Journals/The Journal of Toloo-e-Behdasht_20.docx]

© The Journal of Toloo-e-Behdasht. This work is licensed under CC BY 4.0 (https://creativecommons.org/licenses/by/4.0/).

Volume & Issue: دوره 14 - شماره 1

Title: اثر بخشی درمان شناختی- رفتاری بر کاهش عود اعتیاد به مواد مخدّر در دانشجویان و تأثیر آن بر افزایش انگیزش پیشرفت آنان

http://tbj.ssu.ac.ir/article-1-1623-fa.html

چکیده مقدمه: در مسیر نیل به رضایت، آرامش و شکوفایی نوع بشر مشکلات و موانع متعدّدی وجود دارد. برخی از این موانع ناشی از جهل و نادانی بوده و خودساخته هستند. یکی از مهم‌ترین موانع و مشکلات که سلامت و کارایی نسل فعلی بشر را به شدت به مخاطره انداخته اعتیاد (وابستگی) به مواد مخدّر است. هدف پژوهش حاضر تعیین تأثیر درمان شناختی-رفتاری در افزایش انگیزش پیشرفت دانشجویان وابسته به مواد مخدر وکاهش عود اعتیاد درآنان می باشد. روش بررسی: مطالعه حاضر از نوع نیمه تجربی با پیش آزمون و پس‏آزمون و گروه کنترل می باشد. جامعۀ آماری عبارت ازکلیه دانشجویان معتادی بود که برای درمان و ترک اعتیاد به مراکز درمانی شهر یزد مراجعه می‏کردند. درآغاز،30 نفر ازمیان آنها به صورت تصادفی انتخاب گردیدند و این افراد شامل کسانی می‏شدند که تست مرفین آنها همگی مثبت نشان داده شد. سپس در سه گروه ده نفری شامل گروه آزمایشی، گروه کنترل و گروه بی اثر تقسیم شدند. قبل از اجرای مداخلۀ درمانی همۀ افراد سه گروه به آزمون انگیزش پیشرفت هرمنس پاسخ دادند. بعد از مداخلۀ درمانی نیز افراد سه گروه مجدداً به آزمون انگیزش پیشرفت هرمنس پاسخ دادند. برای تجزیه و تحلیل داده ها از تحلیل واریانس یک راهه و آزمون تعقیبی شفه استفاده گردید. یافته ها: نتایج بر اساس داده های آماری با درجه آزادی 2 و در سطح 001/0=α نشان داد (33/13x^2= )که درمان شناختی-رفتاری، در کاهش میزان عود اعتیاد دانشجویان معتاد مؤثر بوده‏ و توانسته از بازگشت آنها به مصرف مجدد مواد مخدر جلوگیری نماید. نتیجه گیری: در مجموع با توجه به داده های به دست آمده مبنی بر تأثیر مثبت درمان شناختی- رفتاری در کاهش عود بیماران مورد مطالعه و افزایش انگیزش پیشرفت آنان در عرصه های مختلف زندگی از جمله تحصیل و کار توصیه می شود تا از این روش در مراکز درمانی در راستای کاهش عود اعتیاد و افزایش انگیزش پیشرفت توسط افراد مجرب و کار آزموده استفاده گردد. واژه های کلیدی: درمان شناختی- رفتاری، اعتیاد،انگیزش پیشرفت

Title: تأثیر آموزش مبتنی بر نظریه رفتار برنامه‌ریزی شده براتخاذ رفتارهای پیشگیری‌کننده از آرتروز زانودر معلمان زن مقطع ابتدایی

http://tbj.ssu.ac.ir/article-1-1464-fa.html

چکیده مقدمه: بیماری آرتروز از جمله بیماری‌های شایع هم در کشور‌های پیشرفته هم در کشورهای در حال توسعه از جمله ایران است. در مطالعات جمعیتی شیوع آرتروز زانو3/15% در جمعیت بالای 15 سال نشان داده شد. با توجه به مسن‌تر شدن جوامع اهمیت روزافزونی به عنوان یک موضوع بهداشتی عمومی پیدا نموده است. پژوهش حاضر با هدف تعیین تأثیر آموزش بر اساس مدل رفتار برنامه‌ریزی شده بر پیشگیری از آرتروز زانو در معلمان زن مقطع ابتدایی انجام شد. روش‌ بررسی: این مطالعه یک پژوهش مداخله‌ای از نوع نیمه تجربی بود. جامعه مورد مطالعه 114 نفر ازمعلمان زن مقطع ابتدایی بودکه به روش تصادفی وارد مطالعه شدند و به دوگروه مداخله و غیرمداخله تقسیم شدند. پرسشنامه محقق ساخته از طریق خود گزارش دهی تکمیل شد. براساس نتایج اولیه، محتوای آموزشی تدوین و در گروه مداخله ارائه گردید. دوماه بعد از اجرای برنامه آموزشی، پس آزمون انجام شد. داده ها توسط SPSS نسخه 18 تجزیه و تحلیل شد. به دلیل نرمال نبودن توزیع داده‌ها از آزمون‌های ناپارامتریک استفاده گردید. یافته‌ها: سازه‌های تئوری رفتار برنامه ریزی شده (نگرش،هنجارهای ذهنی و کنترل رفتار درک شده) با هم توانستند که 37 درصد قصد و 43 درصد رفتار را پیش‌بینی کنند. در این میان نقش هنجارهای ذهنی (56/0=β)در پیش بینی قصد بیش از سایر سازه‌ها بود. بعد از اجرای برنامه آموزشی میانگین نمره کنترل رفتاری درک شده از 05/4±50/32 به 66/5±82/34 افزایش یافته است. مهمترین موانع اتخاذ رفتارهای پیشگیری از آرتروز زانو" نداشتن فعالیت بدنی منظم"و "عدم استفاده از توالت فرنگی" عنوان گردید. نتیجه‌گیری: مطالعه حاضر اثربخشی برنامه آموزشی مبتنی بر نظریه رفتار برنامه ریزی شده را در پیش بینی قصد و به کارگیری رفتارهای پیشگیری کننده از آرتروز زانو را در معلمان زن تأیید می‌کند.

Title: بررسی سرولوژیک توکسوپلاسموز درزنان مراجعه کننده به آزمایشگاه رفرانس کاشان طی سال های 1390- 1386

http://tbj.ssu.ac.ir/article-1-1465-fa.html

مقدمه:توکسوپلاسموز عفونت مشترک بین انسان و حیوانات است که دارای گستردگی جغرافیایی وسیعی در سطح جهان می باشد.ابتلا خانم ها در سنین کودکی به این بیماری باعث ایجاد مصونیت در برابر ابتلا مجدد در سنین باروری و جلوگیری از عوارض آن خصوصا سقط جنین و توکسوپلاسموز مادرزادی می گردد.این مطالعه به منظورتعیین شیوع آنتی بادی ضد توکسوپلاسما گوندئی به روش سرولوژیک در بین خانم های مراجعه کننده به آزمایشگاه رفرانس کاشان طی سالهای 90-86 انجام شد. روش بررسی: این بررسی یک مطالعه توصیفی است که در آن434 نفر خانم مراجعه کننده به آزمایشگاه رفرانس دانشگاه علوم پزشکی کاشان در طی سالهای 91- 86 مورد مطالعه قرار گرفتند.از خانمهای مذکور 2سی سی خون محیطی گرفته شد و پس از جداسازی سرم، نمونه ها با استفاده از روش الیزا از نظر وجود آنتی بادی های اختصاصی IgG و IgM مورد بررسی و تیتراسیون قرار گرفتند. یافته ها : از مجموع 434 خانم بررسی شده با روش الیزا در این مطالعه311 نفر(66/71%) فاقد آنتی بادی و 123 نفر(34/28%) دارای آنتی بادی اختصاصی ضد توکسوپلاسما از نوع IgGبودند(بیش ازIU/ML 10 ).از این تعداد 7 نفر (6/1%) همزمان دارای آنتی بادی اختصاصی از نوع IgM (بیش از IU/ML 1/1) نیز بودند. توزیع سنی افراد سرم مثبت (IgG+) نشان داد که بیشترین میزان شیوع آنتی بادی با 3/46% در گروه سنی 35- 25و 26% در گروه سنی 45-35 سال بود.6/36% خانم های سرم مثبت از نظر IgG دارای مقدار 50-10 واحد بین المللی آنتی بادی بودند.پنج نفر(1/4%) دارای مقدار آنتی بادی بیش از 250 واحد بودند. نتیجه گیری :در مطالعه حاضر مشخص گردید که در 66/71% از خانم های مراجعه کننده آنتی بادی علیه توکسوپلاسما وجود ندارد که رقم بسیار بالایی است و می توان نتیجه گرفت که درصد زیادی خانمهای سنین باروری در شهر کاشان مستعد ابتلا به عفونت حاد توکسوپلاسموز می باشند بنابراین به منظور پیشگیری از ابتلا به این عفونت بخصوص در دوران بارداری باید آموزشها و توصیه های لازم بهداشتی داده شود.

Title: بررسی تاثیر مداخلات ایمنی مبتنی بررفتار درکاهش اعمال ناایمن در یک شرکت خودروسازی

http://tbj.ssu.ac.ir/article-1-1466-fa.html

چکیده مقدمه: بر اساس مطالعات انجام شده، حدود 90درصد حوادث ناشی از کار در اثر رفتارهای ناایمن و خطای انسانی روی می دهند. لذا در سالهای اخیر، استفاده از تکنیک های ایمنی مبتنی بر رفتار با هدف کاهش حوادث، گسترش زیادی پیدا کرده است. روش بررسی: در این مطالعه نیمه تجربی از نوع قبل و بعد، رفتارهای ناایمن کارگران یک شرکت خودروسازی با استفاده از مشاهده و روش نمونه گیری ایمنی مورد بررسی قرار گرفت. در فاز اول پس از تعریف انواع رفتارهای ناایمن، برای تعیین میزان و انواع آنها، 115کارگر انتخاب و تعداد9200 مشاهده انجام گردید. متغیرهای دموگرافیگ مرتبط با کارگران با استفاده از پرسشنامه جمع آوری گردید. در فاز دوم پس از مدت 3ماه از انجام مداخلات، شامل آموزش کارگران و سرپرستان، نصب علایم ایمنی و استفاده از تنبیه و تشویق، مشاهدات تکرار گردید. یافته ها: یافته های مطالعه نشان داد که در فاز اول رفتارهای ناایمن کارگران 09/13 درصد و در فاز دوم به 04/9 درصد کاهش یافت. از میان این رفتارها، عدم استفاده از وسایل حفاظت فردی و وضعیت ارگونومیکی نامناسب بیشترین فراوانی را دارا بود. میان تعداد رفتارهای ناایمن و سابقه حادثه قبلی (001/0P=) و سطح تحصیلات (04/0P=) ارتباط معناداری مشاهده شد. اما میان تعداد رفتارهای ناایمن با متغیرهای سن، سابقه کار، تعداد افراد تحت تکفل ارتباط معناداری مشاهده نگردید. نتیجه گیری:نتایج مطالعه نشان می دهد توسعه گرایش ایمنی مبتنی بر رفتار، یکی از راهکارهای اصلی کاهش رفتارهای ناایمن و در نتیجه آن کاهش نرخ وقوع حوادث، در صنایع کشور می باشد.

Title: بررسی تاثیر مولفه های فرهنگ سازمانی بر مدیریت دانش در بیمارستان آموزشی درمانی افضلی پور شهر کرمان: 1391

http://tbj.ssu.ac.ir/article-1-1467-fa.html

چکیده مقدمه: مدیریت دانش یکی از دستاوردهای بزرگ عصر اطلاعات و دانش می باشد و تحقیقات نشان می دهد که فرهنگ دانش محور مهمترین عامل در موفقیت دانش است. این تحقیق با هدف بررسی تاثیر مولفه های فرهنگ سازمانی بر مدیریت دانش در بیمارستان های آموزشی درمانی افضلی پور شهر کرمان انجام گرفت. روش بررسی: پژوهش حاضر از نوع تحلیلی بوده و به صورت مقطعی و کاربردی در سال 1391 در بیمارستان آموزشی درمانی افضلی پور شهر کرمان انجام گرفت. جامعه آماری کلیه کارکنان اداری و پاراکلینیک بیمارستان افضلی پور بودند که از بین آن ها 86 نفر به روش نمونه گیری تصادفی ساده انتخاب شدند. داده ها با استفاده از پرسشنامه بسته جمع آوری و با آزمون همبستگی پیرسون و با استفاده از نرم افزار اماری 17spss مورد تحلیل قرار گرفتند. یافته ها: اکثر افراد تحت بررسی در این بیمارستان وضعیت فرهنگ سازمانی را با میانگین نمره 07/3، متوسط ارزیابی کردند. بیشترین امتیاز مربوط به مولفه ی فرهنگ انطباق پذیری با میانگین نمره 18/3 و کمترین امتیاز مربوط به مولفه ی فرهنگ مشارکتی با میانگین نمره 97/2 حاصل شد. مدیریت دانش نیز از سوی پاسخ دهندگان با کسب میانگین نمره (73/2) در وضعیت نامطلوب قرار گرفت. در نهایت رابطه آماری معناداری بین فرهنگ سازمانی و مدیریت دانش با ضریب همبستگی 56/0r= به دست آمد بنابراین با بهبود فرهنگ سازمانی، مدیریت دانش هم بهبود می یابد و همچنین تک تک مولفه های فرهنگ سازمانی نیز بر مدیریت دانش اثر گذارند. نتیجه گیری: رابطه آماری معنادار بین فرهنگ سازمانی و مدیریت دانش، زمینه ساز تغییرات موفقیت آمیز در بیمارستان ها خواهد بود و مدیران را در پیش بینی نظام مند الویت های تغییر و تدوین بهتر استراتژی ها برای اجرای موفقیت آمیز تکنیک ها و فرایند های مدیریت یاری خواهد رساند.

Title: شیوع انگل های روده ای در معلولین ذهنی- جسمی ساکن در مرکز نگهداری شبانه روزی بهزیستی شهرستان تفت

http://tbj.ssu.ac.ir/article-1-1468-fa.html

چکیده مقدمه: شیوع عفونت های انگلی در بسیاری از کشورها بخصوص در کشورهای درحال توسعه به عنوان یک معضل بهداشتی مطرح بوده و بیماریهای انگلی روده ای تاثیر نامطلوبی روی کیفیت زندگی مردم دارند. معلولین ذهنی- جسمی که در موسسات شبانه روزی نگهداری می شوند به دلایل متعددی ازجمله تماس نزدیک با یکدیگر، عدم امکان مراقبت از خود در سطح قابل قبول و نیز عدم رعایت موازین بهداشت فردی در معرض خطر ابتلا به این عفونتها می باشند. هدف از این مطالعه تعیین میزان شیوع انگل های روده ای در جامعه ناتوانان ذهنی مرکز الزهرا شهرستان تفت می باشد. روش بررسی: این مطالعه از نوع توصیفی- مقطعی و به روش نمونه گیری در دسترس بر روی 129 نفر از ناتوانان ذهنی تحت پوشش مرکز بهزیستی شهرستان تفت در تابستان 1393 انجام شد. از هر فرد نمونه مدفوع صبحگاهی تهیه و با روش های لام مستقیم، روش تغلیظی فرمالین- اتر و رنگ آمیزی ذیل نلسون اصلاح شده جهت بررسی انگل های روده ای مورد آزمایش میکروسکوپی قرار گرفت. داده ها با استفاده از بسته نرم افزاری SPSS و با آزمون کای اسکوئر مورد تجزیه و تحلیل آماری قرار گرفتند. یافته ها: بر اساس یافته های بدست آمده در این مطالعه از 129 معلول مورد مطالعه در این بررسی، 1/48 % پسر و بقیه دختر بودند. در این مطالعه در مجموع 55 مورد (6/42%) حداقل به یکی از انگلهای روده ای آلوده بودند. میزان آلودگی به انگلهای روده ای به شرح زیر بودند: کیلوماستیکس مسنیلی 8 مورد( 2/6% )، کیست ژیاردیا لامبلیا 17 مورد(2/13% )، کیست انتاموبا کلی 22 مورد (17%)، بلاستوسیستیس هومینیس 4 مورد (1/3% )، یداموبا بوتچلی 3 مورد(3/2% ) و اندولیماکس نانا 1 مورد(8/0% ). آزمون 2χ نشان داد که توزیع انواع آلودگی های انگلی با متغیر جنس رابطه آماری معنی داری ندارد(05/0 p>). نتیجه گیری: نتایج این تحقیق نشان داد که میزان آلودگی به تک یاخته های روده ای به ویژه برخی گونه های بیماریزا بالاتر از حد انتظار می باشد. تلاش بیشتر برای بهبود و ارتقاء سطح بهداشت و تشخیص به موقع و درمان افراد آلوده می تواند انتقال این عفونتها را در بین جمعیت عقب مانده های ذهنی محدود نماید.

Title: بررسی کارایی فرآیند اکسیداسیون کاتالیتیکی پرتوهای پرانرژی الکترونی توأم با پرسولفات پتاسیم درحذف اسید هیومیک از محلولهای آبی

http://tbj.ssu.ac.ir/article-1-1469-fa.html

مقدمه: مهمترین اثر مواد آلی طبیعی موجود در آب، واکنش با گندزداها و ایجاد محصولات جانبی گندزدایی بوده که اکثراً سرطان‌زا هستند. فرآیندهای اکسیداسیون پیشرفته (AOPs) روشهای متداولی برای حذف مواد آلی از محلول های آبی می‌باشند. کاربرد تابش پرتوهای پرانرژی الکترونی یکی از انواع روش های اکسیداسیون پیشرفته است. لذا در این مطالعه، کارایی فرآیند اکسیداسیون کاتالیتیکی تابش پرتوهای پرانرژی الکترونی توأم با پرسولفات پتاسیم در حذف اسید هیومیک از محلولهای آبی مورد بررسی قرار گرفته است. روش بررسی: این تحقیق یک مطالعه تجربی است که در مقیاس آزمایشگاهی انجام شده است. در این تحقیق، اثر pH (10-4)، غلظت اولیه پرسولفات پتاسیم (mmol/100cc 5/0-1/0)، دوز های جذبی متفاوت پرتوهای پرانرژی الکترونی(kGy 15-1) و غلظت اولیه اسید هیومیک (mg/l 50-10) مورد بررسی قرار گرفت. پرتودهی الکترونی با استفاده از شتاب دهنده الکترون مدل TT200 انجام گرفت و غلظت باقیمانده اسید هیومیک در نمونه ها توسط اسپکتروفتومتر در طول موج 254 نانومتر تعیین گردید. یافته ها: براساس نتایج، تغییر pH دارای تأثیر جزئی بر روی کارایی حذف اسید هیومیک است. بطور میانگین با افزایش pH از 4 به 10، راندمان حذف اسید هیومیک از 59/72% به 36/73% رسیده بود. نتایج نشان داد که با افزایش دوز جذبی از 1 به 15 کیلوگری، کارایی حذف اسید هیومیک افزایش می یابد. بر اساس نتایج با افزایش غلظت پرسولفات، راندمان حذف اسید هیومیک افزایش یافته است بطوری که برای دوز جذبی 3 کیلوگری با افزایش غلظت پرسولفات پتاسیم از mmol/100cc 1/0 به mmol/100cc 5/0، راندمان حذف از 43/69 به 82/83 افزایش یافته بود. آزمایشات سینتیک نشان داد که تجزیه اسیدهیومیک با استفاده از تابش پرتوهای پرانرژی الکترونی از سینتیک درجه دوم پیروی می کند. نتیجه گیری: داده های حاصل از این تحقیق نشان داد که محلول آبی حاوی اسید هیومیک بطور مؤثری توسط پرتوهای پرانرژی الکترونی تجزیه می شود. افزودن پتاسیم پرسولفات قادر به بهبود قابل توجه ای در کارایی فرآیند حذف هیومیک اسید در حضور پرتوهای پرانرژی الکترونی می‌باشد.

Title: بررسی میزان تأثیر آموزش مهارت های مدیریت استرس بر وضعیت اضطراب در معلمین دبیرستان های شهرستان میبد در سال 1391

http://tbj.ssu.ac.ir/article-1-1471-fa.html

مقدمه: استرس یکی از عوامل تاثیر گذار بر عملکرد افراد است که می تواند سلامت آنها را به خطر بیاندازد. شغل معلمی به عنوان یکی از مشاغل استرس زا در جهان شناخته شده است. اضطراب از بیماری های ناشی از استرس می باشد . پژوهش ها نشان می دهد که بین استرس و اضطراب رابطه مستقیم وجود دارد. اضطراب معلم کیفیت تدریس را تحت تاثیر قرار داده و مانع از ارتباط مستمر بین معلم و دانش آموز در حین تدریس می شود. هدف از این مطالعه بررسی تاثیر آموزش مهارتهای مدیریت استرس بر وضعیت اضطراب معلمان بود. روش بررسی: این مطالعه نیمه تجربی بر روی 100 نفر از معلمان 8 دبیرستان(پسرانه/دخترانه) شهرستان میبد که به صورت تصادفی انتخاب شده بودند، انجام شد. ابزار گردآوری اطلاعات پرسشنامه ای با دو بخش اطلاعات دموگرافیک و مقیاس اضطراب موقعیتی- شخصیتی اشپیل برگر بود. برنامه مداخله شامل5 جلسه آموزش مدیریت استرس بود. پیش آزمون قبل از مداخله و پس آزمون دوماه بعد از مداخله انجام شد. داده ها، با استفاده از آزمونهای تی و آنالیز واریانس در نرم افزارSPSS16 ، مورد تجزیه و تحلیل قرار گرفت. یافته ها: میانگین نمره اضطراب شخصیتی معلمان قبل از مداخله 33/7±74/38 (امتیاز کل 80)و بعد از مداخله56/6 ±53/38 و نمره اضطراب موقعیتی آنها قبل از مداخله10/7± 65/40(امتیاز کل 80) و پس از مداخله90/6± 87/38 بود. برنامه آموزشی در کاهش اضطراب شخصیتی معلمان موثر نبود(70/0p=)، اما نمره اضطراب موقعیتی بعد از مداخله معنی دار بود (019/0p=)، بدین صورت که درصد افراد با اضطراب موقعیتی متوسط از 4/34 به 7/28 کاهش یافت. همچنین تاثیر برنامه آموزش مدیریت استرس بر اضطراب موقعیتی و شخصیتی افراد بر حسب سن، جنس، مدرک تحصیلی و سابقه تدریس یکسان بود. نتیجه گیری: نظر به سطح بالای اضطراب در معلمان و ضرورت انجام برنامه های مداخله ای، آموزشهای مدیریت استرس مبتنی بر برنامه در کاهش اضطراب موفق هستند. این برنامه ها که به صورت گام به گام به فرد ارائه می شوند امکان شناخت فرد را از محیط و عوامل استرس زای آن فراهم ساخته و در نهایت میزان استرس و به دنبال آن اضطراب افراد کاهش می یابد.

Title: مقایسه میزان آگاهی و نگرش دندانپزشکان عمومی شهر یزد و دانشجویان سال آخر دندانپزشکی دانشگاه علوم پزشکی شهید صدوقی یزد در مورد بهداشت جیوه در سال1393

http://tbj.ssu.ac.ir/article-1-1472-fa.html

چکیده مقدمه: عدم کنترل صحیح جیوه آلوده کننده مربوط به آمالگام دندانی می تواند با انتشار بخار جیوه در فضای کار و تجاوز از اندازه های استاندارد، مشکلاتی در سلامتی کارکنان دندانپزشکی ایجاد کند. مطالعه حاضر با هدف بررسی میزان آگاهی و نگرش دندانپزشکان عمومی شهر یزد و دانشجویان سال آخر دندانپزشکی دانشگاه علوم پزشکی شهید صدوقی یزد در مورد بهداشت جیوه در سال1393انجام شد. روش بررسی: در این مطالعه توصیفی-مقطعی، پرسشنامه ای (که پایایی و روایی آن از طریق تست های آماری و نظر کارشناسان تایید شد.) بین 87دندانپزشک که به روش نمونه‌گیری‌ ‌تصادفی ساده و 29 دانشجوی سال آخر که به صورت سرشماری انتخاب شده بودند، توزیع گردید. این پرسشنامه شامل سه بخش: اطلاعات دموگرافیک، سوالات مربوط به آگاهی وسوالات مربوط به نگرش نسبت به رعایت بهداشت جیوه در محل کار بود. جهت تجزیه و تحلیل داده‌ها از نرم افزار آماری16 ‌ SPSSو آزمونهای Mann-Whitney وKruskal-Wallis استفاده شد. یافته ها: میانگین نمره آگاهی دندانپزشکان و دانشجویان سال آخر دندانپزشکی به ترتیب 1/2±7/4 و 8/1±7/6 (از 12نمره) بود، که این اختلاف از نظر آماری معنی دار می باشد (0001/0P =). میانگین نمره نگرش دندانپزشکان و دانشجویان سال آخر دندانپزشکی به ترتیب01/2±8/13 و 5/1±9/13 (از 20 نمره) بود، اما این اختلاف از نظر ‌آماری معنی‌دار نبود(088/0P =). نتیجه گیری: نتایج نشان‌داد دندانپزشکان آگاهی و نگرش متوسط و دانشجویان سال آخر دندانپزشکی آگاهی ضعیف و نگرش متوسطی نسبت به رعایت بهداشت جیوه در محل کار خود داشتند.

Title: بررسی دیدگاه دانشجویان در مورد اثرات گروههای دوستی و ورزش برفرایند تعلیم و تربیت

http://tbj.ssu.ac.ir/article-1-1474-fa.html

مقدمه: عوامل محیطی،اجتماعی مانند گروه های دوستی وپرداختن به ورزش میزان لذت را در افراد افزایش داده و انسان را متحول می سازد گروههای دوستی و فعالیت های بدنی و ورزش می توانند عامل موثری در تسریع اجتماعی شدن و تحول روانی،اجتماعی نوجوانان و جوانان باشند.دانشگاه و مدرسه درپیوستن دانشجویان و دانش آموزان به گروه های دوستی و تیم های ورزشی و در نتیجه به عنوان عاملی پویا در رشد و تکامل اجتماعی نقش دارند.هدف این مطالعه بررسی دیدگاه دانشجویان در مورد اثرات گروههای دوستی وورزش برآموزش و یادگیری وبه عبارتی تعلیم و تربیت می باشد. روش بررسی: پژوهش حاضر با استفاده ازروش توصیفی- تحلیلی،مقطعی،در جامعه دانشجویان دانشگاه یزد درسال تحصیلی 91-1390 انجام شده است. به این منظور 406 نفر از دانشجویان به روش تصادفی طبقه ای انتخاب شدند وبه پرسشنامه محقق ساخته ای که روایی وپایایی آن با استفاده از ضریب آلفای کرونباخ 7/82 %در صد محاسبه گردید پاسخ دادند.به منظور توصیف داده هااز شاخص های فراوانی، درصد، میانگین و انحراف معیارو برای آزمون فرضیه ها از آزمون های کای اسکوئر و آزمون همبستگی پیرسون ،آزمون  وآنووا یک طرفه استفاده گردید. یافته ها: نتایج نشان داد 88 در صد دانشجویان معتقد به نقش زیاد وخیلی زیاد تاثیر عامل گروههای دوستی و 2/78 درصد آنهامعتقد به نقش زیاد وخیلی زیاد تاثیر عامل ورزش بر روند رشد تعلیم و تربیت می باشند که تاثیر عوامل مذکور از نظر آماری معنادار می باشند(01p ≤). از بین متغیرهای دموگرافیک فقط وضعیت تاهل با گروههای دوستی و ورزش و شغل مادر با گروه دوستی رابطه معنی داری دارند ودر هیچکدام دیگر از متغیرها با عوامل اجتماعی مذکور رابطه معنی داری مشاهده نگردید. نتیجه گیری:دیدگاه دانشجویان نشان داد عوامل گروههای دوستی و ورزش بر تعلیم و تربیت تاثیر گذاری بسیار بالایی داشته که نهایتا به بهبود روابط اجتماعی می انجامدو امکان یادگیری را در گروههای متفاوت افزایش می دهد و تقویت گروههای دوستی و تیم های ورزشی در محیطهای آموزشی تاثیر بسیار مهم در اجتماعی نمودن نوجوانان و جوانان دارد که تسهیل کننده کسب مهارت های زندگی وفرایند یادگیری است و در نتیجه موجب رشد تعلیم و تربیت افراد جامعه می گردد.

Title: مدل سازی تغییرات زمانی و مکانی کیفیت آب زیرزمینی در مناطق خشک با استفاده از روش‌های زمین آماری (مطالعه موردی: دشت کوهپایه – سگزی اصفهان)

http://tbj.ssu.ac.ir/article-1-1473-fa.html

چکیده مقدمه: منابع آب زیرزمینی به عنوان یکی از مهمترین منابع آبی در مناطق خشک و نیمه خشک، محسوب می‌شوند، از این‌رو حفاظت کیفی آنها از اهمیت زیادی برخوردار است. از طرفی پایش و بررسی کیفیت آب زیرزمینی مشکل و هزینه‌بر می‌باشد، لذا استفاده از روش های درون‌یابی، ضرورری به نظر می‌رسد. روش بررسی: در این تحقیق توصیفی، تغییرات مکانی و زمانی برخی از پارامترهای کیفی آب زیرزمینی دشت کوهپایه-سگزی اصفهان شامل Ec، TDS و Na در دوره آماری 1389-1374، مورد بررسی قرار گرفته و میزان و جهت تغییرات این پارامترها، مشخص شده است. بدین منظور ابتدا چهارروش زمین آماری و قطعی قابل اجرا در محیط 3/9 ArcGISیعنی کریجینگ، کوکریجینگ، تابع شعاع محور و عکس فاصله وزنی، مورد بررسی قرار گرفت. سپس بهترین تابع و توان برای هر یک از چهار روش ذکر شده و بهترین روش برای هر پارامتر در هر سال دوره آماری بر اساس کمترین میزان ریشه میانگین مربعات خطا (RMSE)، انتخاب گردید. در نهایت نقشه‌های پهنه‌بندی پارامترها در محیط 3/9ArcGIS ، ترسیم گردید. یافته‌ها: نتایج به دست آمده نشان می‌دهد که برای هر سال و هر پارامتر روشی خاصی با تابع یا توانی خاص، بهترین نتیجه را دارد و روند منظمی در آنها وجود ندارد، . اما در سال های بعد از سال 1383، روش های کریجینگ و کوکریجینگ، نتایج بهتری نسبت به دیگر روش‌ها داشته‌اند. نتیجه‌گیری: نتایج این بررسی نشان می‌دهد که کیفیت منابع آبی دشت کوهپایه - سگزی در سال 1389 نسبت به سال 1374، کاهش چشمگیری یافته است. بیشترین میزان تغییرات نیز در مناطق نزدیک به رودخانه زاینده‌رود رخ داده است به طوریکه در مناطق جنوبی دشت کانون‌های بحرانی ظاهر شده است، که این خود گواه بر نقش مؤثر این رودخانه بر سفره های مسیر خود می باشد.

Title: بررسی فرسودگی شغلی در پرستاران بخش‌های ICU، اورژانس و جراحی بیمارستان‌های آموزشی علوم پزشکی مازندران و ارتباط آن با تنیدگی ادراک شده

http://tbj.ssu.ac.ir/article-1-1475-fa.html

چکیده مقدمه: حرفه پرستاری تنیدگی زاست و فشار روانی آن می‌تواند منجر به فرسودگی شغلی شود اما ادراک متفاوت از یک رویداد تنیدگی زا می تواند این رابطه را تعدیل کند. هدف پژوهش بررسی فرسودگی شغلی پرستاران بخش‌های ICU ، اورژانس و جراحی و ارتباط آن با تنیدگی ادراک شده است. روش بررسی : پژوهش توصیفی تحلیلی و مقطعی است. جامعه پژوهش کلیه پرستاران زن و مرد شاغل در بخش‌های ICU ، اورژانس و جراحی چهار بیمارستان آموزشی دانشگاه علوم پزشکی مازندران در شهر ساری بودند (180=N). نمونه بر اساس جدول کرجسی ومورگان 120 نفر تعیین شد که از پرستاران بخش-های ICU، اورژانس و جراحی به شیوه نمونه‌گیری تصادفی طبقه‌ای انتخاب شدند. ابزار پژوهش پرسشنامه‌های فرسودگی شغلی و تنیدگی ادراک شده بودند. جهت تحلیل داده‌ها از تحلیل واریانس یکراهه، ضریب همبستگی پیرسون و تحلیل رگرسیون همزمان استفاده شد. یافته‌ها: نتایج نشان داد تفاوت معناداری بین فرسودگی شغلی پرستاران بخش‌هایICU، اورژانس وجراحی وجود ندارد (683/1,F= 191/0P=). بین فرسودگی شغلی و ادراک مثبت از تنیدگی رابطه منفی و معنادار(307/0R= - ،001/ 0P=)، بین فرسودگی شغلی و ادراک منفی از تنیدگی رابطه مثبت و معنادار(404/0 R= ،000/ 0P=) وجود داشت. همچنین ادراک منفی از تنیدگی بالاترین سهم (333/0β=) را در تبیین فرسودگی شغلی داشت. نتیجه‌گیری: شیوه‌های ارزیابی فرد نقش کلیدی در تعدیل تنش‌های تجربه شده در محیط کار را دارد لذا شناسایی افرادی که ادراک منفی از تنیدگی‌های شغلی دارند به منظور تغییر ارزیابی منفی آنان از موقعیت تنیدگی‌زا می‌تواند در کاهش فرسودگی شغلی و افزایش کیفیت خدمات پرستاری ارائه شده موثر باشد.
